# Diaminopyrene modified reduced graphene oxide as a novel electrode material for excellent performance supercapacitors†

**DOI:** 10.1039/c9ra10429a

**Published:** 2020-01-08

**Authors:** Hongwei Kang, Chengpeng Zhang, Yonggui Xu, Weiyang Zhang, Jianhua Jiao, Zhikun Li, LeiLei Zhu, Xiaoqian Liu

**Affiliations:** Henan Key Laboratory of Nanocomposite and Application, Institute of Nanostructured Functional Materials, Huanghe Science and Technology College Zhengzhou 450006 China hongweikang@infm.hhstu.edu.cn zhikunli@infm.hhstu.edu.cn; Henan Science and Technology Exchange Center with Foreign Countries China

## Abstract

In this paper, novel reduced graphene oxide (rGO) composites (DAPrGOs) modified by diaminopyrene (DAP) were successfully synthesized *via* a facile solvothermal reaction method and used for supercapacitors. Compared with the pristine rGO, the DAPrGO1 electrode showed distinctly better performance (397.63 F g^−1^*vs.* 80.29 F g^−1^ of pristine rGO at 0.5 A g^−1^) with small charge transfer resistance. When a symmetric device was fabricated using DAPrGO1 as the active material, it also exhibited a high capacitance of 82.70 F g^−1^ at 0.5 A g^−1^ with an energy density of 25.84 W h kg^−1^ at a power density of 375 W kg^−1^, and even offered a high power density of 7500 W kg^−1^ (18.71 W h kg^−1^) at 10 A g^−1^. Moreover, the device possessed good electrochemical stability up to 20 000 cycles, implying promising applications in energy storage fields.

## Abstract 2

Schematic illustration of the facile synthesis process of DAPrGOs nanocomposites, Ragone plots and the superior cyclic stability of the assembled DAPrGO1//DAPrGO1 SSS.

## Graphical abstract

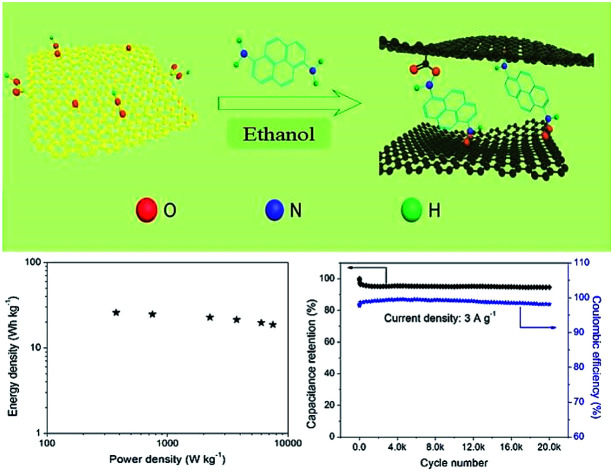

## Introduction

With the ever-growing demand to use non-fossil energy sources and reduce greenhouse gases, supercapacitors have captured considerable attention as green and sustainable energy storage devices; they can deliver electrical energy very quickly, thus giving high power density and are indispensable supplements to batteries. In spite of the many achievements that have been made recently, factors like relative low specific capacitance, poorer energy density than batteries and insufficient cycle stability, still limit the applicability of supercapacitors in daily life.^1–3^

The core problem of supercapacitors is electroactive materials, and the utilization of high-powered redox materials is a versatile solution to upgrade the performance of supercapacitors. In this sense, kinds of redox active potential materials, representatives of which are inorganic metal oxides,^4–7^ conductive organic materials,^8–11^ and carbon materials^12–14^ are successfully used to enhance the pseudocapacitance effect of the device.

In the exploration of high-performance and supercapacitor, the 2D carbon sheet-graphene, featured with high electron conductivity, is an indispensable element.^15–17^ Graphene offers many advantages over other conventional conducting materials, including easily acquired by mature methods, large surface area, flexibility and electrochemical stability, providing a reliable way to fabricate wearable devices. However, the capacitance of the pristine graphene is relatively low for lacking electroactive groups. In this perspective, modification of graphene nanosheet using pseudocapacitive materials aforementioned is highly needed and turns out to be very effectively.^18–22^ Among these various graphene based composites, we are particularly interested in using aromatic amine compound to modify graphene and improve the comprehensive properties of composite materials, in consideration that the active COOH group, epoxy groups of graphene oxide will readily react with amine to form stable covalent bond. Therefore, aromatic amines with high electrochemical activity, such as aniline (PANI), phenylenediamine and aminopyrene have been chosen as promising materials to join into graphene matrix by covalent modification to fabricate the graphene–amine composites. These composites could fulfill superior pseudocapacitance of organic molecules and the excellent conductivity of graphene, and thus to bring in high capacitance and high stability as well as the overall electrochemical performance of supercapacitors eventually.^23–27^

Therefore, as a kind of polycyclic aromatic hydrocarbon with amino group, diaminopyrene can form a good synergistic effect with graphene, which is a potential electrode material in the field of energy storage. In this work, we report the synthesis of 1,6-diaminopyrene (DAP) modified reduced graphene oxide materials (DAPrGOs) for the application of supercapacitors in alkaline electrolyte. Compared with pristine rGO, DAPrGOs show an obviously better performance for the redox active pyrene units and carboxyl groups. The optimum DAPrGO1 electrode exhibits a high specific capacitance of 397.63 F g^−1^ at 0.5 A g^−1^, and even at high current density of 10 A g^−1^, it still has a capacitance of 224.52 F g^−1^. When a symmetric supercapacitor device are fabricated using DAPrGO1 as active material (named as DAPrGO1//DAPrGO1 SSS), the SSS exhibits an energy density of 25.84 W h kg^−1^ (375 W kg^−1^) at 0.5 A g^−1^, and even offers a power density of 7500 W kg^−1^ (18.71 W h kg^−1^) at 10 A g^−1^. Under common atmosphere, the device still possesses fine electrochemical stability with capacitance retention of 94.57% after 20 000 cycles, showing its superiority as electrode material in energy storage field.

## Experimental

### Synthesis of DAP materials

**Scheme 1:**
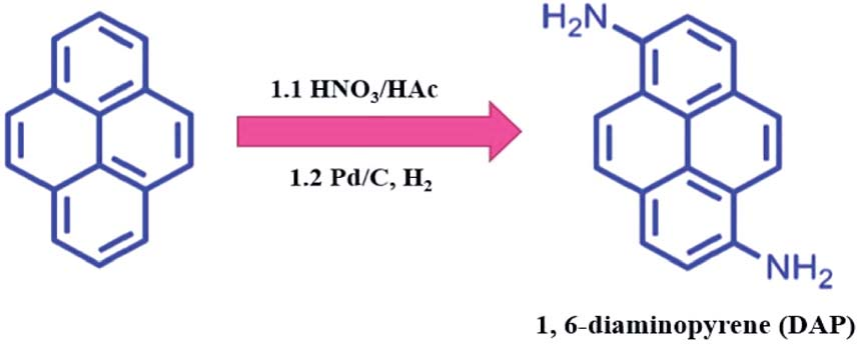
Schematic illustration of the synthesis process of DAP.

As shown in Scheme 1, DAP was prepared by a modified two-step routine from pyrene.^28^ In detail, 10.0 g of pyrene was dissolved in 100 mL of acetic acid at 90 °C, and then 7.5 mL of HNO_3_ (65%) was added to the above solution for 5 min. After the mixture was stirred for 30 min, the yellow crude product was filtrated (a mixture of 1,3-dinitropyrene, 1,6-dinitropyrene and 1,8-dinitropyrene). Moreover, when half of the crude product was dissolved in 200 mL of ethanol, 550 mg of 10% Pd/C was added to the solution at room temperature, and reacted with hydrogen gas for 24 h. After that, the reaction mixture was purified by column chromatography using AcOEt/DCM (v/v, 1 : 9) to afford 820 mg of DAP (overall yield: 14.3%) (Fig. S1 and S2†). ^1^H NMR (400 MHz, CDCl_3_) *δ* 7.93–7.81 (m, 4H), 7.69 (d, *J* = 9.2 Hz, 2H), 7.34 (d, *J* = 8.1 Hz, 2H), 4.39 (s, 4H).

### Synthesis of DAPrGOs composites and rGO

**Scheme 2:**
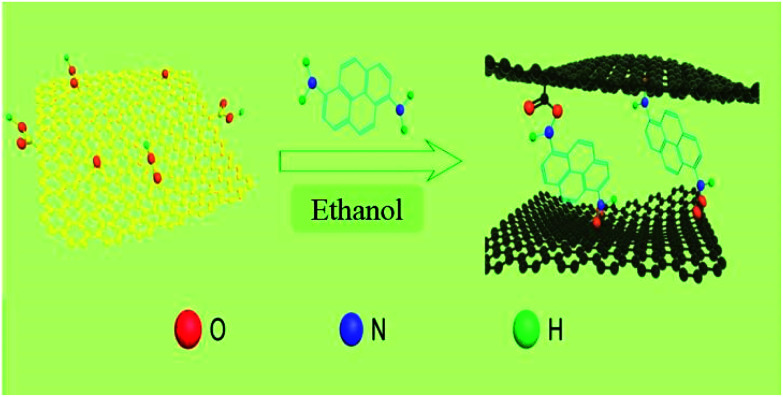
Schematic illustration of the synthesis process of DAPrGOs composites.

GO was prepared by the modified Hummer's method.^29^ In a typical experiment, 80 mg of GO was dispersed in 80 mL of ethanol and sonicated for 1 h, and then 80 mg of DAP was added to the GO dispersion. When it stirred vigorously for 3 h, the mixed solution was poured into a Teflon-lined stainless steel autoclave (50 mL) for solvothermal reaction at 160 °C for 15 h. After the mixture was cooled to room temperature, the composite was collected by filtration and washed with ethanol to remove the residual reagents that it not effectively involved in the reaction. At last, the product was obtained as black powder after drying in a vacuum oven at 70 °C for 12 h. The final obtained composites named as DAPrGO1 (according to the mass ratio of DAP to GO is 1 : 1). When 40 mg and 160 mg of DAP was added to the GO solution, the synthesized composites was named as DAPrGO0.5 and DAPrGO2, respectively. The facile synthesis process of DAPrGOs composites as illustrated in Scheme 2.

The preparation process of rGO material is similar to that of the DAPrGO samples except that there is no DAP material.

### Material characterizations

DAP was identified by 400 M NMR spectrometer (Bruker ADVANCE). The as-prepared materials were characterized by scanning electron microscopy (SEM, Quanta 250 FEI), transmission electron microscope (TEM, JEOL JEM-2100), X-ray diffraction diffractometer (XRD, Bruker D8 Advance) with Cu Kα radiation (*λ* = 1.5417 Å), Raman microscope (Raman, Renishaw inVia) employing a 633 nm laser excitation (50% laser power), Fourier transform infrared (FTIR, Thermo Scientific Nicolet iS5), thermal gravimetric analysis (TGA, SDT Q600) under N_2_ atmosphere with a heating rate of 2 °C min^−1^, and Brunauer–Emmett–Teller measurement (BET, Micrometric ASAP 2020 system).

### Electrochemical measurements

The prepared samples were used as the active materials, polytetrafluoroethylene (PTFE, 60%) and conductive carbon black was used as binder and conductive additive, respectively. The weight ratio of active materials, PTFE and conductive carbon black was 8 : 1 : 1. When the mixture was stirred evenly in isopropanol and evaporated to form a homogeneous paste, the slurry was evenly coated on foam nickel and extruded together. Cut out the sample which is not pressed on the foam nickel and placed it in the drying oven, and vacuum drying at 70 °C for 12 h. Each electrode was loaded about 1.5–2.0 mg (area density is about 2.0 mg cm^−2^), and electrochemical tests were performed in 2 M KOH aqueous solution.

In the three-electrode system, the prepared electrode, a platinum foil, and Ag/AgCl electrode were served as working electrode, counter electrode, and reference electrode, respectively. The capacitance was calculated according to the following eqn (1) based on GCD curves:1
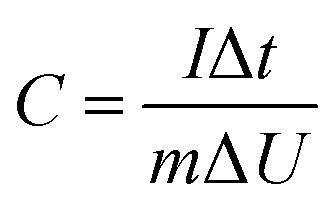
where *C* (F g^−1^) is the specific capacitance, *I* (A) is the response current, Δ*t* (s) is the discharge time, *m* (g) is the mass of active material, Δ*U* (V) is the range of potential.

In this simple symmetric supercapacitors system, two electrodes were separated by two layers of cellulose film, sandwiched in two pieces of plastic, and nipped by a binder clip. The SSS was immersed in 2 M KOH aqueous solution overnight for electrochemical testing. The specific capacitance (*C*, F g^−1^), energy density (*E*) and power density (*P*) of the SSS were calculated according to the following eqn (2)–(4), respectively:2
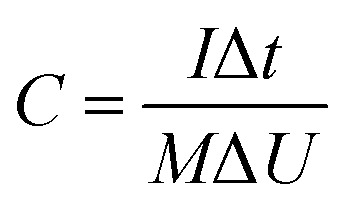
3
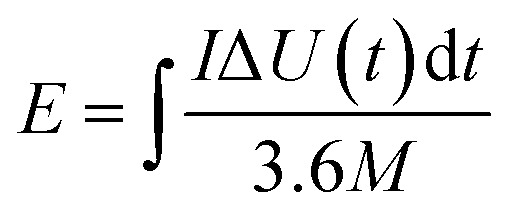
4
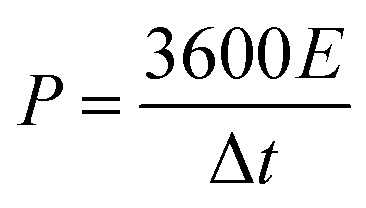
where *I* (A) is the response current, Δ*t* (s) is the discharge time, Δ*U* (V) is the range of potential, *M* (g) is the total mass of both electrodes.

## Result and discussion

**Fig. 1:**
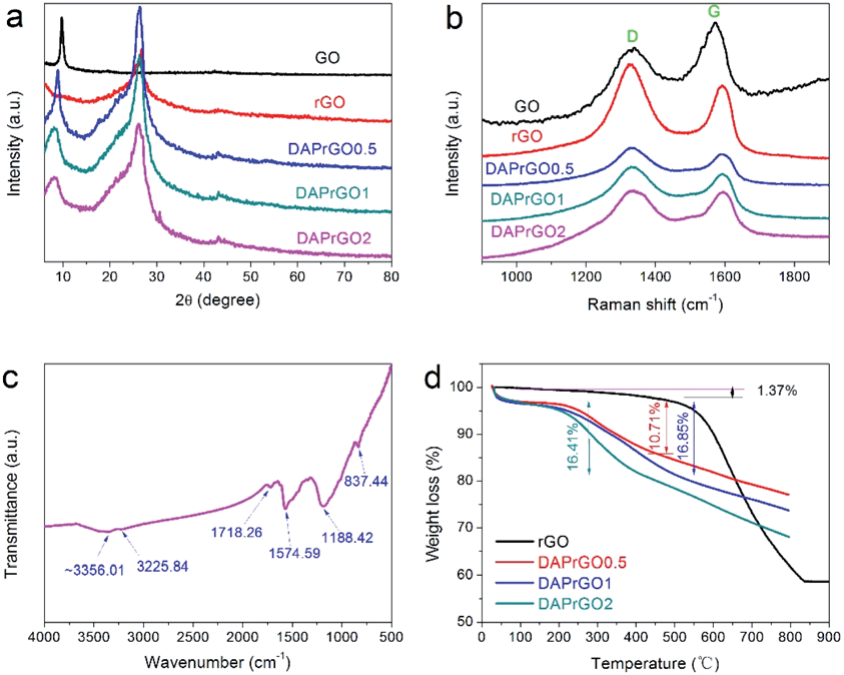
(a) XRD patterns and (b) Raman spectra of GO, rGO, DAPrGO0.5, DAPrGO1, and DAPrGO2 samples; (c) FTIR spectrum of DAPrGO1 sample; (d) TGA curves of rGO, DAPrGO0.5, DAPrGO1, and DAPrGO2.

To determine the crystalline structure, crystallinity and chemical components, the XRD patterns of the as-prepared GO, rGO, DAPrGO0.5, DAPrGO1, and DAPrGO2 samples were presented in Fig. 1a. It can be found that there is only one distinct broad peak located at around 26.2° and a very weak peak located at around 43.8° in XRD pattern of rGO, which indicates that the GO was well reduced to rGO by solvothermal reaction. However, when organic DAP was added into the reaction, GO was not completely reduced, which indicated that the presence of DAP affected the reduction of GO. Simultaneously, it meant that the synthesized DAPrGOs composites contain both GO and rGO materials. In addition, we can also observed that the diffraction peak intensity of DAPrGOs samples at 26.2° is significantly higher than that of rGO materials, which indicates that the composites have a better crystallinity. The crystallinity and structural characteristics of the GO, rGO, DAPrGO0.5, DAPrGO1, and DAPrGO2 materials were further detected by Raman spectra, as exhibited in Fig. 1b. It can be clearly observed that the typical characteristic peaks are located at around 1337 cm^−1^ and 1591.78 cm^−1^, corresponding to the D-band (a representation of the structural defects and unordered carbon of the graphitic structure) and G-band (originated from the E_2g_ phonon in-plane vibration of sp^2^-bonded carbon atoms), respectively.^29–32^ The intensity ratio of *I*_D_/*I*_G_ was usually used to evaluate the degree of defects and graphitization of carbonaceous materials. The calculated *I*_D_/*I*_G_ values of all prepared samples were 0.86 (GO), 1.25 (rGO), 1.15 (DAPrGO0.5), 1.11 (DAPrGO1), and 1.02 (DAPrGO2). Compared with GO, the increase in the ratio of *I*_D_/*I*_G_ for DAPrGOs composites indicated that more defects produced after solvothermal reduction.

The FTIR spectra of GO, rGO, DAPrGO0.5, DAPrGO1, and DAPrGO2 samples were used to further confirm the functional groups in these prepared materials. As presented in Fig. 1c, several major peaks can be observed in the spectrum of DAPrGO1 sample. The absorption peaks located at around 837.44 cm^−1^, 1188.42 cm^−1^, 1574.59 cm^−1^, and 1718.26 cm^−1^ could be attributed to the N–H wagging vibration, C–N stretching vibration, N–H bending vibration (it mainly comes from amide bond and the diaminopyrene which are not bonded with graphene functional group), and C–O stretching vibrations which is due to the p–π conjugation of the non-shared electron pair on the N atom in the amide I band with the carbonyl group, respectively.^30,33,34^ What's more, combined with the comparison of the spectra of DAP, rGO, and DAPrGOs (Fig. S3†), we infer that the broad peak located at around 3356.01 cm^−1^ and a weak peak located in 3225.84 cm^−1^ were mainly attributed to the –NH stretching vibration (some –NH bonds of them should come from the formed amide bond) and –NH_2_ stretching vibration, as well as a small amount of the stretching vibration of –OH functional groups which not reduced in rGO. It was noteworthy that one of the most obvious absorption peak located at around 1050 cm^−1^ in rGO materials is not obvious in the composites. We inferred that the content of this functional group is greatly reduced by the participation of organic DAP in the reaction. These results indicated that the organic DAP is successfully synthesized in the rGO materials after solvothermal reaction reduction.

The thermal stability of rGO and all DAPrGOs samples were investigated and their TGA curves were shown in Fig. 1d. It can be clearly observed that there are three major steps of weight loss in these TGA curves of DAPrGOs samples: below ∼100 °C, around 100–480 °C and around 480–800 °C. The rapid weight loss below ∼100 °C was mainly attributed to the removal of adsorbed water (∼3.5% weight loss). And the weight loss around 100–480 °C was mainly due to the steam escape from organic DAP materials existed in rGO layers, the DAP without effective participation from the DAPrGOs materials, and the decomposition of unstable oxygen-containing groups in GO/rGO materials. And combined with the analysis of TGA curve of rGO, we conclude that the mass content of organic molecule DAP in the DAPrGOs composite is about 10–17%. The weight loss above 480 °C was mainly attributed to the cleavage and decomposition of stable oxygen-containing groups and other functionalities groups, as well as the combustion of some graphite carbon (∼10% weight loss).^35,36^ It was noteworthy that when the temperature rises slowly to 800 °C, the weight loss of the DAPrGOs composites is only about 23–32%. And combined with the TGA curve of rGO materials, it can be inferred that the modification of rGO by DAP molecule enhances the thermal stability of the composite materials.

**Fig. 2:**
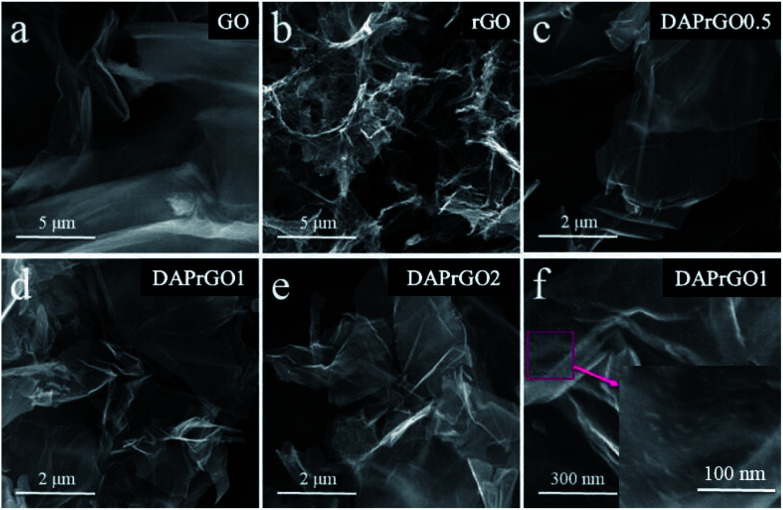
Low-magnification SEM images of (a) GO, (b) rGO, (c) DAPrGO0.5, (d) DAPrGO1, (e) DAPrGO2, and (f) high-magnification SEM image of DAPrGO1.

**Fig. 3:**
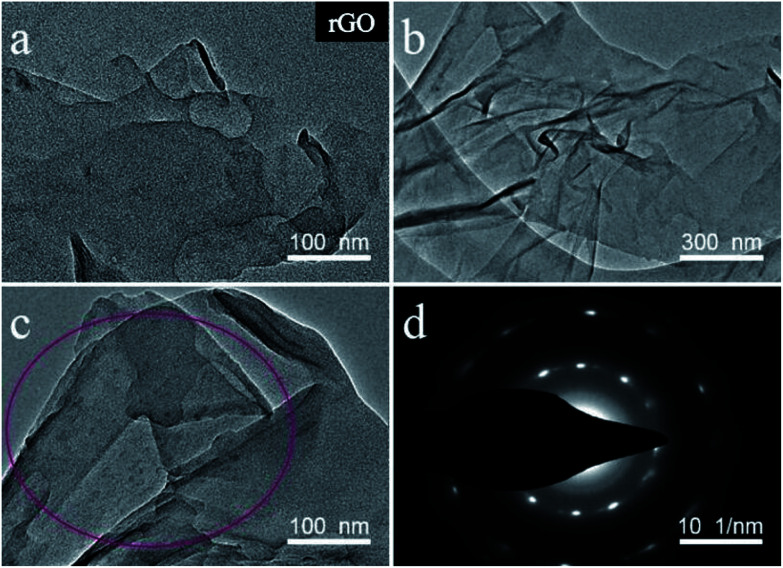
TEM images of (a) rGO and (b and c) DAPrGO1; (d) SAED image of DAPrGO1.

We investigated the morphologies and nanostructures of the prepared samples by using SEM and TEM. The SEM images of GO, rGO, DAPrGO0.5, DAPrGO1, and DAPrGO2 were shown in Fig. 2. The GO materials displayed an obvious agglomeration phenomenon, but after solvothermal reduction, the rGO showed a rich loose porous nanostructure, and the agglomeration and stacking of rGO sheets were obviously reduced (Fig. 2a and b). DAPrGOs composites synthesized by adding different reactant DAP contents were shown in Fig. 2c–e. It can be observed that all SEM images show a gauze-like transparent structure, suggesting that the material synthesized by reduction with the participation of DAP material has a fluffy lamellar structure. These structures were conducive to shortening the diffusion distance of electrolyte ions and facilitating the rapid transport of ions. The high resolution SEM image and the enlarged view of pink area of DAPrGO1 material in Fig. 2f clearly showed that the small DAP molecules with a small amount of agglomeration are uniformly dispersed on the rGO lamellae. The interior microstructures of samples were further characterized by transmission electron microscope (TEM). As shown in Fig. 3a, it displayed a large number of staggered rGO sheets with few layers, and there was no obvious attachment on the rGO sheets. Fig. 3b and c showed the TEM images of the synthesized sample DAPrGO1. It can be clearly seen that the composite exhibits a transparent lamellar structure with a small amount of wrinkles. More noteworthy was that a large number of additives, organic DAP materials, are evenly attached on the surface of rGO and between the rGO lamellae, which indicates that DAP materials are well dispersed in rGO material. What's more, the selected-area electron diffraction (SAED) pattern of DAPrGO1 materials with a well-defined rings indicated that a porous nanostructure and with a good crystallinity.^37,38^ These porous nanostructures with thin layers not only facilitated the diffusion and transmission of ions, but also provided more storage sites and reaction sites, which have great advantages and potential in the field of energy storage.

**Fig. 4:**
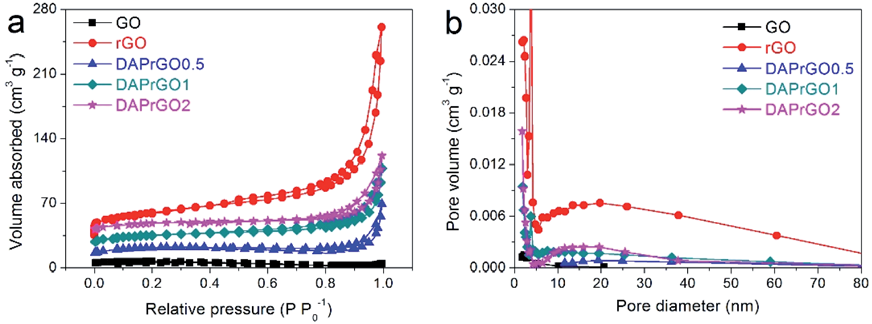
(a) N_2_ adsorption–desorption isotherms, and (b) the pore size distribution curves of GO, rGO, DAPrGO0.5, DAPrGO1, and DAPrGO2 samples.

The typical N_2_ adsorption–desorption isotherms of GO, rGO, DAPrGO0.5, DAPrGO1, and DAPrGO2 samples were illustrated in Fig. 4a. All samples displayed a typical type-IV isotherm with a hysteresis loop at *P*/*P*_0_ of about 0.45–0.65, indicating the existence of mesoporous and macropores structure. The hierarchical porosity composed of mesopores connected with macropores is very conducive to the buffer and diffusion of electrolyte, and reduces the volume change during the charge–discharge cycling, improving the cycle stability.^39–41^ The corresponding pore size distribution calculated by the Barrett–Joyner–Halenda (BJH) method were shown in Fig. 4b. It can be observed that the size of pore diameter of DAPrGO1 and DAPrGO2 samples is mainly concentrated in the 7–35 nm region, and that of DAPrGO0.5 sample is mainly concentrated in the 13–50 nm region. In addition, we can also observed that the pore size of GO materials is mostly less than 10 nm, and the pore size and total pore volume increased obviously after reduction of GO materials. The BET surface area (*S*_BET_) and textural properties of the GO, rGO and DAPrGOs samples prepared by us were summarized in Table S1.† We can found that the *S*_BET_ of DAPrGOs composite is much higher than that of material GO materials, which indicates that the degree of agglomeration of rGO obtained by solvothermal reduction with the participation of organic small molecule DAP is obviously reduced. Moreover, the pore size and total pore volume of DAPrGOs composites were also much higher than that of material GO. In addition, a comparison between the DAPrGOs materials can be seen that the DAPrGO1 sample has an optimal *S*_BET_ (38.83 m^2^ g^−1^) and pore volume (0.129 cm^3^ g^−1^), which is more conducive to the storage of ions benefiting from more contact/reaction sites. It is worth noting that the *S*_BET_ and pore volume of DAPrGOs composite are significantly smaller than that of rGO, which is mainly due to the existence of organic DAP molecules. It not only increases the interaction force with rGO molecules, but also forms amide bond with the functional groups in rGO, thus reducing the layer spacing and porosity, which is inevitable in graphene-based organic composites.^29,42,43^

**Fig. 5:**
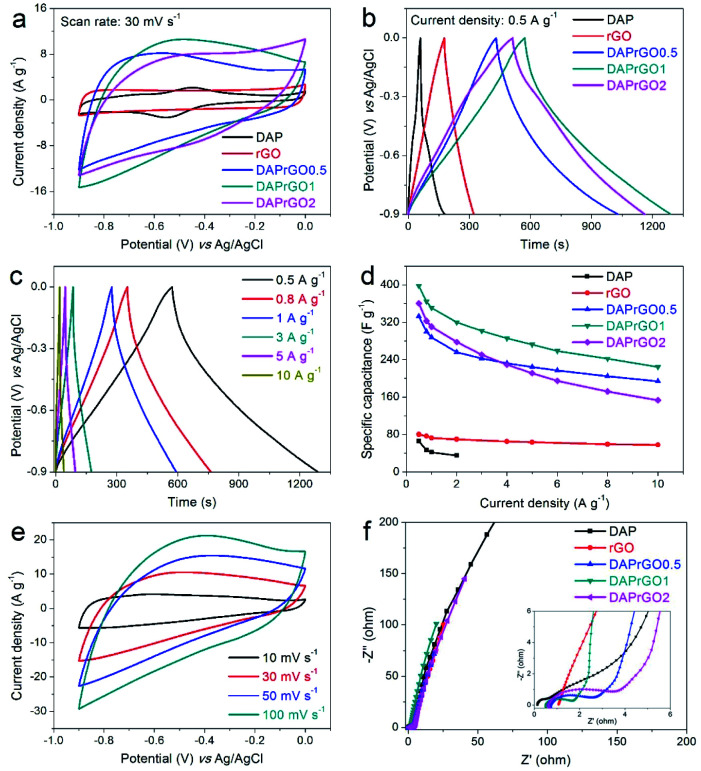
(a and b) The comparison of electrochemical properties of the DAP, rGO, DAPrGO0.5, DAPrGO1, and DAPrGO2 electrodes; (c) GCD curves of DAPrGO1 electrode at different current densities; (d) specific capacitance *vs.* current density for all kinds of prepared electrodes; (e) CV curves at various scan rate for DAPrGO1 electrode; (f) Nyquist plots.

In this paper, the electrochemical performances of the prepared electrodes were firstly investigated in a conventional three-electrode system by using an alkaline medium (2 M KOH) as the electrolyte. As illustrated in Fig. 5(a and b), by analyzing the capacitance performance of the electrode and comparing the CV curves with the GCD curves, we can clearly observed that the electrochemical performance of the composite electrode material is significantly improved, and the DAPrGO1 electrode has the best capacitance. What's more, the wide redox band in CV curve and the non-linear curve in GCD curve indicated the generation of Faraday capacitance. Further calculated from the GCD curves, the DAP, rGO, DAPrGO0.5, DAPrGO1, and DAPrGO2 electrodes exhibited a specific capacitances of 65.83, 80.29, 332.68, 397.63, and 360.38 F g^−1^ at a current density of 0.5 A g^−1^, respectively. Compared with DAP electrode and rGO electrode, the capacitance of DAPrGO1 electrode was almost 6.04 times and 4.95 times respectively. Fig. 5c showed the GCD curves of DAPrGO1 electrode at various current densities from 0.5 to 10 A g^−1^. All GCD curves exhibited a non-linear symmetrical triangle, which indicates that the DAPrGO1 electrode contains both double layer capacitance and Faraday capacitance. In addition, the almost non-existent IR drop on the discharge curve indicated that the electrode material has a small internal resistance, showing the characteristics of a new excellent electrode material in the field of energy storage.^44^ The relationship between specific capacitances and current densities of all electrodes were shown in Fig. 5d. It can be directly observed that even at high current density of 10 A g^−1^, the composite electrodes still had a high capacitance, which indicates that it has excellent rate capability. It was important to note that with the current density exceeding 4 A g^−1^, the capacitance of DAPrGO0.5 electrode material is better than that of DAPrGO2 electrode material, which means that the capacitance of DAPrGO2 electrode material decreases more seriously with the increase of current density. We inferred that this result is due to the high content of organic DAP material in DAPrGO2 composites which affects the structural stability and rate capability. The bulge peaks present in the CV curves at various scan rate for DAPrGO1 electrode in Fig. 5e indicated the redox reaction present during charge and discharge, which means the existence of pseudocapacitance.

To investigate the electrode conductivities and resistance properties of all prepared electrodes, the electrochemical impedance spectroscopy (EIS) tests were conducted at a frequency range of 0.01 Hz to 100 kHz. It was well known that the closer the straight line in the low frequency region is to the *X*-axis, the closer the capacitance behavior is to the ideal capacitor. Therefore, from Fig. 5f we can found that all the electrodes we prepared exhibit approximately ideal capacitive behavior except for DAP electrode. However, the intersection of the curve with the *X*-axis of DAP electrode was smaller than that of other electrode, especially rGO electrode, which indicates that the DAP electrode has a better wettability and compatibility with alkaline medium electrolytes.^45^ In addition, a smaller semicircular arc diameter in the high frequency region indicated that the DAPrGO1 electrode in all composite electrodes has a smaller interfacial transfer resistance, meaning a higher ionic conductivity.^46^ Therefore, DAPrGOs composite electrodes not only made up for the disadvantage of low capacitance behavior of DAP electrode, but also enhanced the compatibility and wettability of rGO electrodes with KOH electrolyte as much as possible, thus improving the comprehensive performance of electrode materials.

**Fig. 6:**
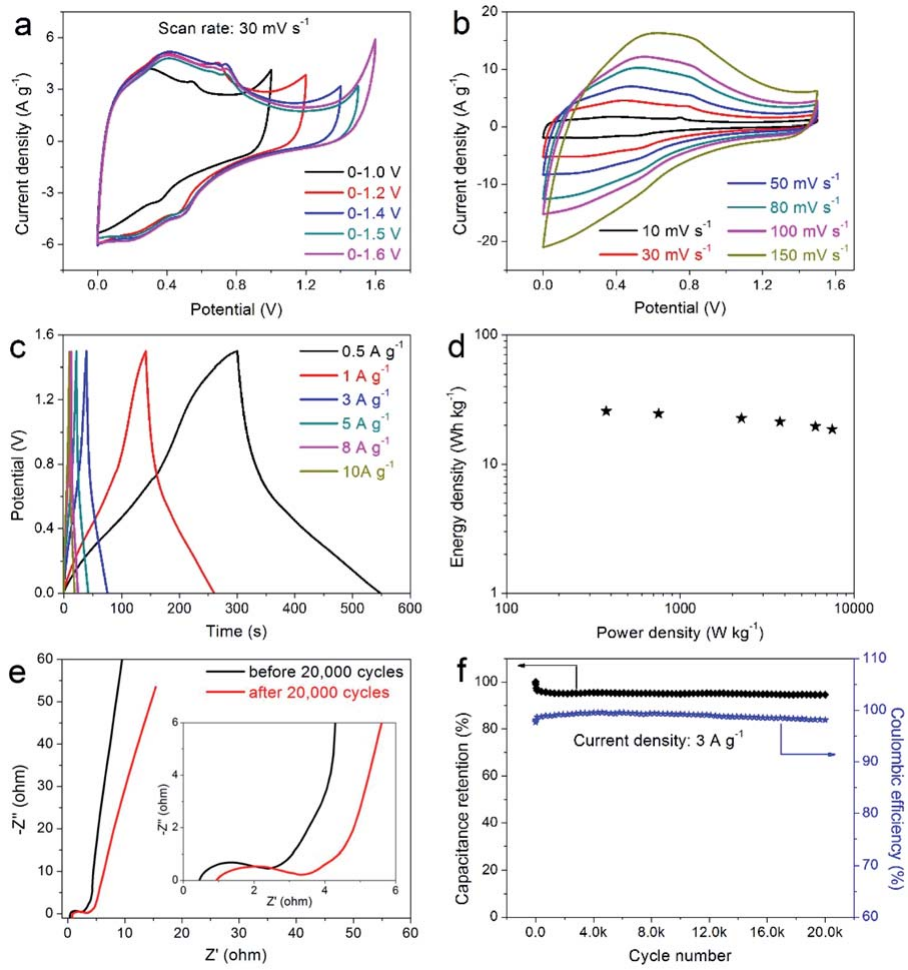
(a) CV curves of the DAPrGO1//DAPrGO1 symmetric supercapacitor system (SSS) at different potential windows; (b) CV curves, (c) GCD curves, (d) Ragone plot, (e) Nyquist plots, and (f) cyclic stability and coulombic efficiency of DAPrGO1//DAPrGO1 SSS.

In order to further verify the practicability of the prepared composite electrode and its potential application in the field of supercapacitors, the prepared DAPrGO1 electrode was assembled to a symmetric supercapacitor system (SSS) with 2 KOH as electrolyte. By comparison, we chose a potential window of 0–1.5 V because this potential range effectively contains the Faraday reaction without polarization (Fig. 6a). The CV curves at various scan rates shown in Fig. 6b displayed a similar shape, indicating an excellent reversibility. What's more, these curves also showed that the reaction process includes the characteristics of double-layer capacitance and pseudocapacitance. As the potential gradually increased from the low potential, the response corresponding to the current gradually changed from the electric double layer reaction to the Faraday reaction, and then to the final electric double layer reaction. Fig. 6c showed the GCD curves of DAPrGO1//DAPrGO1 SSS at different current densities. It was calculated that the specific capacitance of the SSS is 82.70 F g^−1^ at 0.5 A g^−1^, and it still had a specific capacitance of 59.87 F g^−1^ even up to 10 A g^−1^, exhibiting an excellent rate capability.

The energy and power densities of the DAPrGO1//DAPrGO1 SSS were calculated based on the eqn (3) and (4) and the Ragone plot were shown in Fig. 6d. The DAPrGO1//DAPrGO1 SSS delivered a superior energy density of 25.84 and 18.71 W h kg^−1^ at a power density of 375 and 7500 W kg^−1^, respectively. The cycle life of our assembled DAPrGO1//DAPrGO1 SSS was measured at the current density of 3 A g^−1^ for 20 000 cycles to further examine its actual application performance. As shown in Fig. 6f, it can be observed that the capacitance after multiple charging and discharging cycles almost does not decay, except that the initial 800 cycles attenuate with a relatively large amount of content. And after 20 000 cycles, the SSS presented an excellent cyclic stability with 94.57% capacitance retention of initial capacitance. In addition, an excellent Coulomb efficiency over 97% was maintained during 20 000 cycles, and the EIS data of the DAPrGO1//DAPrGO1 SSS without larger changes before and after 20 000 cycles further proved its outstanding cycle life (Fig. 6e), which is mainly due to the stability of ion/charge transport channels in composites. These high energy and power densities, as well as excellent cyclic stability, were also reflected in DAPrGO0.5//DAPrGO0.5 SSS and DAPrGO2//DAPrGO2 SSS (Fig. S4a and b†). The great improvement of electrochemical properties of composites was mainly attributed to the synergistic effect of organic DAP and rGO materials, which not only gives full play to their respective advantages, even promoted their respective advantages, but also avoids the impact of disadvantages as far as possible, thus gaining the enhancement of comprehensive properties of composites. The composite electrode material with excellent electrochemical properties was equivalent or superior to the similar electrode materials reported previously (Table S2†).

## Conclusions

In summary, novel interesting DAPrGO composites were successfully synthesized through a simple solvothermal method and used for supercapacitors. The results showed that the composite materials exhibited more excellent performances compared with pristine rGO and organic small molecule DAP materials. The optimum DAPrGO1 electrode exhibited a high specific capacitance of 397.63 F g^−1^ at 0.5 A g^−1^. When a symmetric device was fabricated using DAPrGO1 composites as active material, it exhibited a device capacitance of 82.70 F g^−1^ with an energy density of 25.84 W h kg^−1^ at 0.5 A g^−1^, and even offered a power density of 7500 W kg^−1^ at 10 A g^−1^. The SSS possessed excellent stability with capacitance retention of 94.57% after 20 000 cycles under common atmosphere. These outstanding properties demonstrated that DAP is a very promising candidate to composite with rGO for the application of high-performance supercapacitors.

## Conflicts of interest

There are no conflicts to declare.

## Supplementary Material

RA-010-C9RA10429A-s001
