# Supplementary material for: Diaminopyrene modified reduced graphene oxide as a novel electrode material for excellent performance supercapacitors
Source: RSC Adv. 2020 Jan 8;10(3):1507–13. doi: 10.1039/c9ra10429a (PMC9047509; doi:10.1039/c9ra10429a)
Supplement: RA-010-C9RA10429A-s001 [file RA-010-C9RA10429A-s001.pdf]

Electronic Supplementary Material (ESI) for RSC Advances.
This journal is © The Royal Society of Chemistry 2020

Diaminopyrene modified reduced graphene oxide as novel electrode material for excellent performance supercapacitors

Hongwei Kang[a]*, Chengpeng Zhang[b], Yonggui Xu[b], Weiyang Zhang[a], Jianhua Jiao[b], Zhikun Li[a]*, LeiLei Zhu[b], and Xiaoqian Liu[b]

[a]Henan Key Laboratory of Nanocomposite and Application, Institute of Nanostructured Functional Materials, Huanghe Science and Technology College, Zhengzhou 450006, China.
[b]Henan Science and Technology Exchange Center with Foreign Countries.

*Corresponding Authors.

E-mail: hongweikang@infm.hhstu.edu.cn, *zhikunli@infm.hhstu.edu.cn*

Supplementary Figures

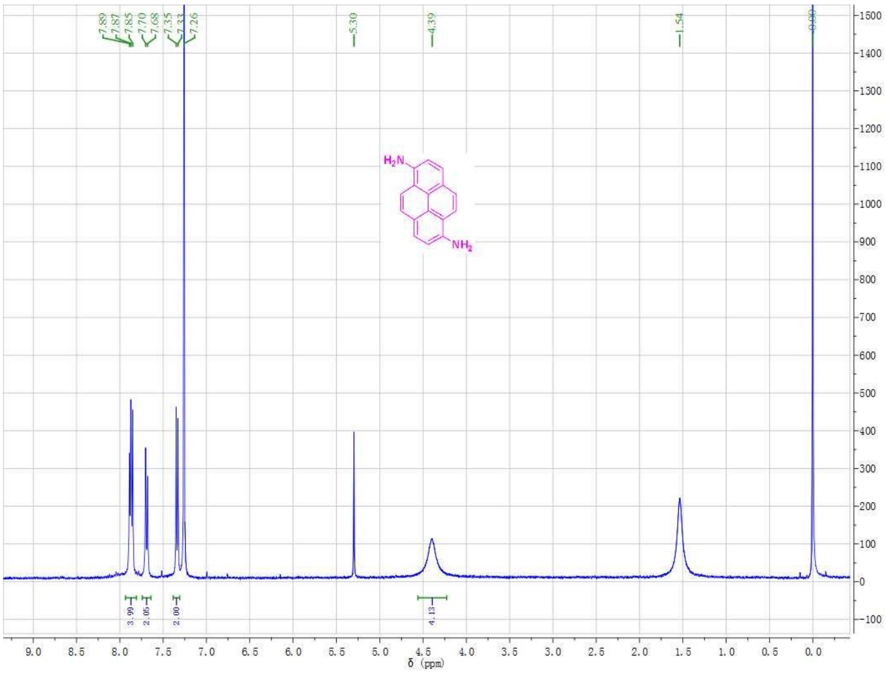

Fig. S1 NMR spectra of the prepared DAP materials.

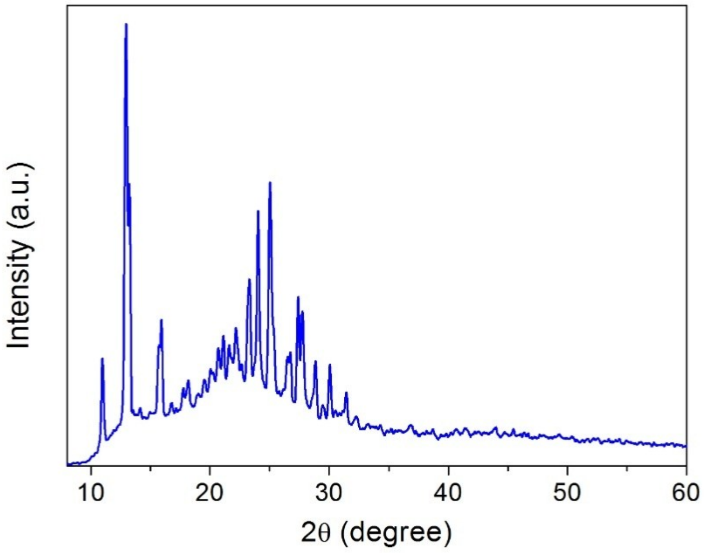

Fig. S2 XRD pattern of DAP sample.

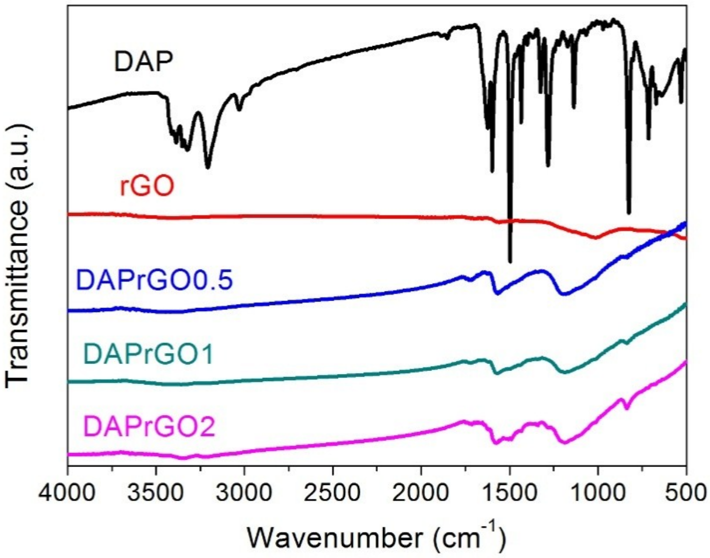

Fig. S3 The comparison of FTIR spectra of DAP, rGO, DAPrGO0.5, DAPrGO1, and DAPrGO2 samples.

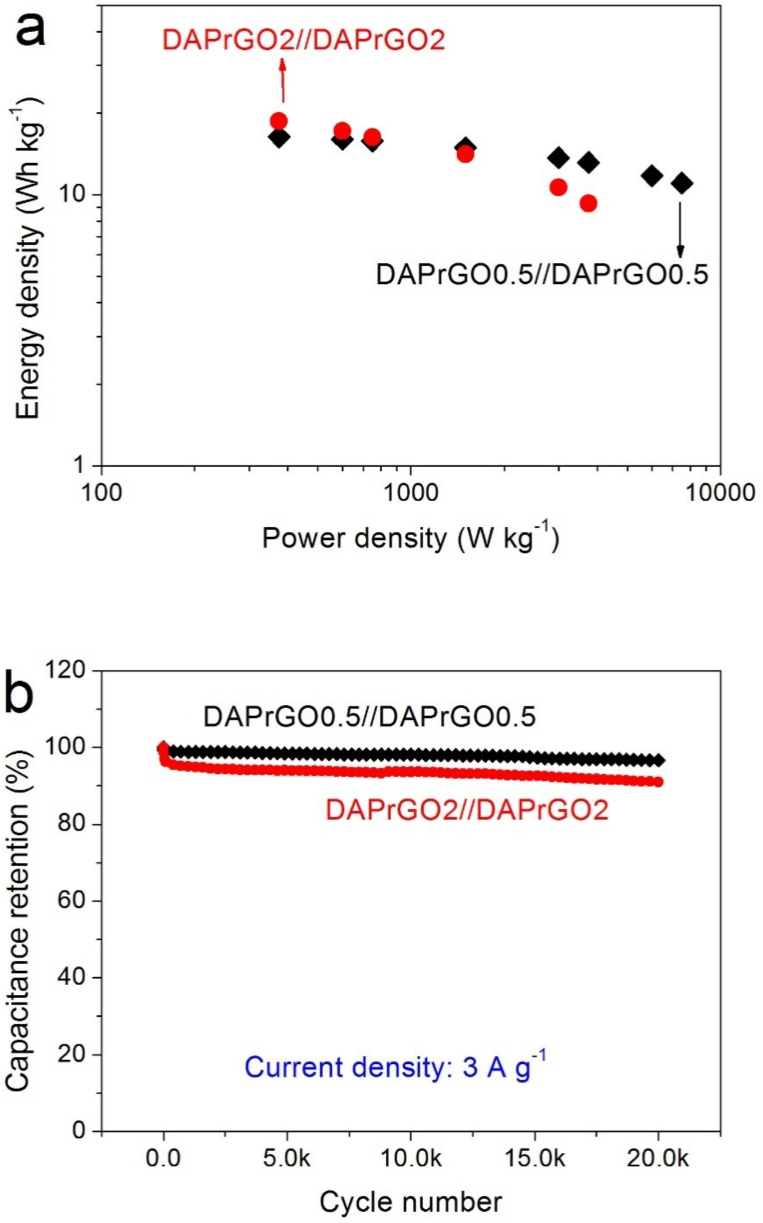

Fig. S4 (a) Ragone plot and (b) Cyclic stability of DAPrGO0.5//DAPrGO0.5 SSS and DAPrGO2//DAPrGO2 SSS.

Table S1 Textural parameters of the GO, rGO and DAPrGOs samples

Sample	S_{BET} (m^2 g^{-1})	D_{DFT} (nm)	V (cm^3 g^{-1})
GO	5.80	2.199	0.002
rGO	122.96	14.592	0.531
DAPrGO0.5	26.56	40.321	0.084
DAPrGO1	38.83	13.963	0.129
DAPrGO2	32.17	12.730	0.126

Note: S_{BET} is the BET surface area; D_{DFT} is the DFT desorption average pore diameter; V is the total pore volume.

Table S2 Comparison of electrochemical performances for the similar conductive organic-based electrode materials in the previous literatures.

Samples	*Sc* ($F\ g^{-1}$)	Current density (Scan rate)	E ($W\ h\ kg^{-1}$) P ($W\ kg^{-1}$)	capacitance retention (cycle numbers)	Ref.
AQS@rGO	567.1	$1\ A\ g^{-1}$	29.2; 3420	83.1% 10,000	1
quinone-coated carbon onions	264	$1.3\ A\ g^{-1}$	4.5	97% 10,000	2
PPy-RGO	255.7	$0.2\ A\ g^{-1}$	7.02; 89	93% 1,000	3
Ap-rGO	160	$5\ mV\ s^{-1}$	5.6; ~ 50	85% (5,000)	4
RGO/BPA	466	$1\ A\ g^{-1}$	/; /	90% (4,000)	5
PYT-NH_2/rGO	326.6	$0.5\ A\ g^{-1}$	15.4; 300.3	~100% (25,000)	6
RGO/UCNTs/PANI	359.3	$1\ A\ g^{-1}$	7.4; 189.0	80.5% (2,000)	7
AQSGH	258	$0.3\ A\ g^{-1}$	/; /	>100% (2,000)	8
PPD modifie rGO	316.54	$10\ mV\ s^{-1}$	27.01; 926.06	93.66% (4000)	9
PF-PAM-RGO	214.8	$0.2\ mA\ cm^2$	80.59 µWh cm^2; 2549.83 µW cm^2	92.36% 8000	10
DAPrGO1	397.63	$0.5\ A\ g^{-1}$	25.84 (375); 18.71 (7500)	94.57% (20,000)	This work

Notes and references

1 R. Y. Shi, C. P. Han, H. Duan, L. Xu, D. Zhou, H. F. Li, J. Q. Li, F. Y. Kang, B. H. Li and G. X. Wang, Adv. Energy Mater., 2018, **8,** 1802088.
2 D. M. Anjos, J. K. Mcdonough, E. Perre, G. M. Brown, S. H. Overbury, Y. Gogotsi and V. Presser, Nano Energy, 2013, **2,** 702-712.
3 J. B. Zhu, Y. L. Xu, J. Wang, J. P. Wang, Y. Bai and X. F. Du, Phys. Chem. Chem. Phys., 2015, **17,** 19885-19894.
4 E. Y. L. Teo, N. L. Hong, R. Jose and K. F. Chong, Rsc Adv., 2015, **5,** 38111-38116.
5 H. X. Hu, Z. A. Hu, X. Y. Ren, Y. Y. Yang, R. B. Qiang, N. An and H. Y. Wu, Chinese J. Chem., 2015, **33,** 199-206.
6 J. Q. Shi, Z. P. Zhao, J. C. Wu, Y. B. Yu, Z. K. Peng, B. J. Li, Y. S. Liu, H. W. Kang and Z. Y. Liu, ACS Sustain. Chem. Eng., 2018, **6,** 4729-4738.
7 Y. P. Huang, J. J. Zhou, N. Gao, Z. X. Yin, H. H. Zhou, X. Y. Yang and Y. F. Kuang, Electrochim. Acta, 2018, **269,** 649-656.
8 Q. Wu, Y. Y. Sun, H. Bai and G. Q. Shi, Phys. Chem. Chem. Phys., 2011, **13,** 11193-11198.
9 X. N. Lu, L. Y. Li, B. Song, K. S. Moon, N. N. Hu, G. L. Liao, T. L. Shi and C. P. Wong, Nano Energy, 2015, **17,** 160-170.
10 M. Y. Jia, L. L. Cui, F. Peng, Y. Li, L. S. Xu and X. J. Jin, New J Chem., 2019, **43,** 6394-6403.
